# The national alcohol helpline in Sweden: an evaluation of its first year

**DOI:** 10.1186/1747-597X-9-28

**Published:** 2014-07-11

**Authors:** Kozma Ahacic, Lena Nederfeldt, Ásgeir R Helgason

**Affiliations:** 1 Department of Public Health Sciences, Karolinska Institutet, Tomtebodavägen 18 A, 171 77 Stockholm, Sweden; 2 Centre for Epidemiology and Community Medicine, Stockholm, Stockholm County Council, Sweden; 3 Reykjavik University, Reykjavik, Iceland

**Keywords:** Quitline, Helpline, Alcohol, AUDIT, Treatment, Counseling

## Abstract

**Background:**

Telephone helplines are easily available and can offer anonymity. Alcohol helplines may be a potential gateway to a more advanced support protocol, and they may function as a primary support option for some. However, although telephone helplines (quitlines) make up an established evidence-based support arena for smoking cessation, few studies have described such telephone-based alcohol counseling.

**Methods:**

This study describes the basic characteristics of callers (n = 480) to the Swedish Alcohol Helpline during its first year of operation, and assesses aspects of change in alcohol behavior in a selected cohort of clients (n = 40) willing to abstain from anonymity and enter a proactive support protocol.

**Results:**

During the study period, 50% of callers called for consultation regarding their own alcohol use (clients), a third called about relatives with alcohol problems, and the others called for information. The clients’ average age was 49 years, and half were females. The clients’ average AUDIT score at baseline was 21 (std. dev. =7.2). Approximately a quarter had scores indicating hazardous alcohol use at baseline, while the others had higher scores. In a follow-up pilot study, the average AUDIT score had decreased from 21 to 14. While clients reporting more severe alcohol use showed a significant decrease at follow-up, hazardous users exhibited no change during the study period.

**Conclusion:**

The study indicates that telephone helplines addressing the general public can be a primary-care option to reduce risky alcohol use. A randomized controlled study is needed to control for the effect of spontaneous recovery.

## Introduction

Heavy or risky alcohol use is a threat to public health as it is causally linked to a wide range of harms and diseases [1]. Screening and performing a brief intervention is an evidence-based and cost-effective strategy for the early management of alcohol-use disorder [2-7]. Hazardous use of alcohol can be regarded as a level of consumption that entails a risk of harmful use of alcohol or alcohol dependence in the future.

Telephone helplines addressing the general public are well-established enterprises, e.g. for smoking cessation. The worldwide success of telephone helplines in relation to smoking cessation [8,9] suggests that a comparable approach to the risky use of alcohol may be a viable option. Many studies have found telephone-based support to be a successful and cost effective method of treatment, such as in the aftercare of substance abusers [10-27]. However, judging from the scientific studies describing telephone-based alcohol counseling, the Swedish Alcohol Helpline seems to be among the first to offer such a service as a primary-care option for risky users of alcohol.

Although people with alcohol dependence, the most severe state of alcohol-related disorder, may require specialized treatment [28,29], brief clinically based counseling episodes have been found to be an effective way of reducing consumption and risk among people with hazardous alcohol consumption [30-32].

About 14 percent of the Swedish population have been estimated to have hazardous alcohol consumption [33]. Hazardous use of alcohol is usually identified by screening instruments, which measure alcohol consumption and its consequences for the individual’s physical and mental health [34]. The more severe states, harmful use of alcohol and alcohol dependence, are indicated by several behavioral, cognitive, and physiological symptoms. They are generally detected in clinical interviews, but brief and straightforward proxies for clinical interviewing are also provided in questionnaires [35]. WHO makes different recommendations according to the severity of the disorder [35,36]. For hazardous users simple advice suffices, but for harmful use WHO recommends not only simple advice but also brief counseling and continued monitoring. Alcohol dependence should prompt a referral to specialists for diagnostic evaluation and treatment.

Typically, the most severe state, i.e., dependence, is characterized by a strong desire to consume alcohol, impaired control of its use, persistent drinking despite harmful consequences, greater priority given to drinking than to other activities and obligations, increased tolerance of alcohol, and physical withdrawal reactions when alcohol use is discontinued. Not all hazardous drinkers become dependent over time. But once dependency on alcohol develops, it may become more difficult to stop or decrease consumption. Nonetheless, even if people with alcohol dependence are at a greater risk of incurring high levels of harm, the bulk of harmful consequences is to be found among people with hazardous use of alcohol, simply because of their greater number – the so-called prevention paradox [37].

Any telephone helpline is reliant on people’s own identification of their behavior as a problem. Whether hazardous alcohol users spontaneously seek help to change their behavior from a service like the telephone helpline is not clear. Telephone support offers anonymity and is easy accessible; even when ill and in bed, you can always make a call. On the other hand, people may prefer face-to-face contact [38], and the telephone can be a problematic option for some, e.g., for persons with a speech or hearing impairment. There may also be other disadvantages, such as the demands placed on existing infrastructure, and a lack of guidelines. There are methodological concerns in relation to previous research on helplines, counselor fatigue, etc.

Alternative techniques for improving early detection, such as screening or incorporating relevant questions into clinical interviews in health care have been recommended [39]. But there have also been obstacles to the implementation of these methods, such as lack of knowledge and skills, lack of time, and financial disincentives [2]. Moreover, not all people visit primary care. The threshold for actively seeking help from a telephone helpline, where you can choose to be anonymous, is likely to be lower than in primary care.

The Swedish Alcohol Helpline is a nationwide service operated by the Stockholm County Health Service in collaboration with Karolinska Institutet. Due to the success of quitlines for smoking cessation in high-income countries, including Sweden [8,9], it was decided to develop and test a comparable approach to the risky use of alcohol. Together with several other organizations, Stockholm County Council started to operate a smoking quitline in 1998 [40,41]. The “know how” and the techniques (computer systems, logistics, and many other factors) from the tobacco quitline were applied to the development of the Alcohol Helpline.

The Swedish Alcohol Helpline, a nation-wide service, has been in operation since January 2007. Its principal objective has been to encourage people contemplating change in their alcohol drinking habits, but it also offers help in sustaining change and preventing relapse among persons who have already decreased their alcohol consumption.

The primary aim of the present study is to describe callers to the alcohol helpline and, in particular, to assess the characteristics of callers requesting counseling. This involves considering distributions by gender, age, and alcohol use (i.e., non-hazardous use, hazardous use, harmful use, and dependence). A secondary aim, in a pilot study, is to follow up a sample of callers who enrolled for more intensive (proactive) support to assess their goals, perceived successes, and possible changes in alcohol use over time.

## Methods

### The alcohol helpline

Computerized client records and a treatment protocol that had previously been developed for the tobacco quitline were adapted for alcohol counseling [42,43], and personnel were trained accordingly. The Helpline opened in January 2007 and has been in operation since then. Information about the services was spread in information campaigns. During the first year, information was mainly aimed at people in Stockholm County, but from 2008 onwards efforts made were nationwide.

Both “reactive” and “proactive” services are provided by the Helpline. *Reactive services* are ones where clients signing up for support are encouraged to call the Helpline whenever needed. The client can remain anonymous and the counselor will not initiate any contact. *Proactive services* are ones where all clients signing up for treatment are offered a call-up facility at one or several pre-arranged time. There were no rules to qualify for the proactive services, except that proactive clients had to leave their name and telephone number to enable the Helpline to initiate contact with them.

### Developing the counseling services

The theoretical base for the counseling practiced at the Helpline can be characterized as involving a mixture of Motivational Interviewing (MI) and Cognitive Behavior Therapy (CBT). Most counselors had previous work experience from the County’s Prevention Center, and had various degrees of education and counseling experience in the field. Their training program at the helpline had four main pillars. The counselors were given: a) a background to alcohol and addiction treatment and epidemiology; b) basic training in the use of elementary CBT tools, covering the principles of negative and positive reinforcement, classical and operant conditioning, gradual exposure, basic behavior-analysis procedures (such as identifying cues for craving); c) comprehensive training in MI, including supervision based on MITI.3 feedback from the Motivational Interviewing Coding Laboratory (MIC-Lab) at the Karolinska Institutet; and, d) training in the use of telephone and computer-based client support and documentation systems.

The counselors were trained by qualified MI and CBT therapists, researchers, physicians, and nurses with a background in alcohol and drug rehabilitation services. After an initial training period with professional actors, the counselors were instructed to tape-record real-life treatment sessions after obtaining the informed consent of their clients. All counselors had to achieve an acceptable level in MI [44] before starting to work independently with clients. Later, the counselors were also offered individual feedback and group coaching six times a year concerning both the MI and CBT aspects of the counseling. The main emphasis was on maintaining and enhancing counselors’ competence in MI and on integrating CBT methods into MI.

Adherence to the MI treatment protocol was checked at regular intervals in tape-recorded treatment sessions. The coding was performed by trained personnel at an external coding laboratory (the MIC-Lab). The MIC-Lab also provided continuous feedback to the counselors during and after their initial MI training [45].

### The treatment protocol

Individually tailored treatment plans were established and documented during the first call from the clients and developed throughout the treatment period. A client call normally lasted for about 20 minutes, but the first call was usually longer – around 45 minutes – due to the need for initial data collection for client records. There was no time restraint. The treatment plans included a formulation of the client’s primary and secondary aims, and their strategies for behavioral change. It also included a behavioral analysis, focusing on assessment of alcohol habits. Most treatment was delivered over the phone. Exceptionally, however, behavior assessments, such as AUDIT [36], were mailed to the client, who completed the form and returned it to helpline staff. Clients and relatives were also offered complementary self-help material, including booklets with practical exercises and guidance, which otherwise was transmitted over the phone. The booklets included an alcohol-drinking diary, a list of different strategies for reducing drinking at parties with exercises concerned with the setting-up of different goals and identifying obstacles, and also a general exercise for identifying relevant situations and ways of saying no to alcohol in each one.

Each assessment was discussed and feedback given in the first call, or – when an assessment had been mailed – in a subsequent call to the Helpline. In the counseling different behavior-related issues were explored: how prepared the clients were for behavioral change; their belief in their own ability to change; and, their related worries and ambivalences. The counselors also answered questions that the client had about other alcohol-related matters. If needed, clients were referred to clinical specialists. Accordingly, the telephone discussions often touched on treatment issues, such as the need for medical support, and also upon the social situations of the clients.

### Material

*Sample 1* included all persons (n = 480) calling the helpline during its first phase of operations, from May to December 2007. Those who expressed a willingness to receive support in changing their alcohol consumption were recognized as clients (n = 226). Following WHO’s recommendations [35,36], persons seeking support for their own problems but who were assessed by the counselors to have problems that were too severe for brief counseling were referred directly to clinical specialists. These persons (n = 16) were regarded as ineligible for outcome assessment and excluded at baseline.

*Sample 2 – the pilot study*: After 6 months, all clients who had completed baseline screening were considered for inclusion in a follow-up study. Only those who, during their first call, had chosen a proactive service, and thereby left their telephone number and consent to be contacted, could be included. Reactive clients were not asked whether they wanted to participate because priority was given to anonymity. The follow-up included questions about treatment goals and client satisfaction. The alcohol-behavior assessment instrument used, AUDIT [36], was introduced three months after the Helpline started. Some clients had therefore not been initially assessed, including some of the proactive clients who were eligible for follow-up. Of 79 proactive clients, relevant information regarding AUDIT score at baseline and contact information was available for only 57, who comprised the study population for the pilot study. Of these 57 clients, 40 (70%) could be located for a follow-up interview.

The study design, with follow-up of clients of the Alcohol Helpline, was approved by the local ethical committee in Stockholm (diary number: 2008/2022-31).

### AUDIT

For the assessment of severity of alcohol consumption, the Alcohol Use Disorders Identification Test (AUDIT) was used [33,46-49]. It comprises 10 items in three domains: recent alcohol use, alcohol-dependence symptoms, and alcohol-related problems. Responses to each item are scored 0–4 points, and the total score ranges from 0 to 40 points, where higher scores indicate more hazardous alcohol use [36]. An AUDIT score can be used to detect hazardous use, harmful use, and dependence. Customary cut-offs for hazardous use in Sweden are 8 points for men, and 6 for women [50], but alternative cut-offs have been recommended to differentiate between individuals in populations with a high use of alcohol [33,47]. Use of the alternative cut-offs results in four categories [35,36,51]. The first category (scores 0–8) indicates low-risk drinking or abstinence, the second (scores 9–15) hazardous use, the third (scores 16–19) harmful use, and the fourth (score 20–40) dependence.

### Other variables

During their initial contact with the Helpline, all clients were asked to formulate primary personal goals. What the clients wanted to achieve by contacting the Helpline are represented by the broad themes emerging from a qualitative analysis of the answers to this open-ended question. At follow-up, perceived success in goal achievement was assessed on a scale from 1 to 10, where 1 corresponded to not at all and 10 to completely.

The follow-up questions by phone included: “Have you received any support other than from the Alcohol Helpline? Suggested response options were: no support; a family member; a friend/friends; a friend/friends at work/the boss; Alcoholics Anonymous (AA); Länkarna (a Swedish counterpart to AA); health care personnel, alcohol-related primary care reception or in-patient care; other professional help. Another questionnaire item was: “Have you used any pharmaceutical drug for alcohol dependence? Suggested response options were: “Have not used any drugs: Antabus; Campral; Revia; Others, which…” The questionnaire also covered the dates between which the different drugs were used. Responses to the above items were collapsed into a dichotomous variable “professional help or medicines, yes or no” to facilitate overview and analysis, i.e., to get a reasonably even distribution between the categories. Later, to discriminate between different kinds of help, further distinctions were made in the regression analysis: professional help, yes or no; medication, yes or no; help from a friend or friends, yes or no; help from the family, yes or no.

Similarly, to get a reasonably even distribution across categories, age was divided into four groups: young (15–39 years); middle-aged (40–49 years); young-olds (50–64 years); and, old-olds (65+).

### Analysis

Independent sample t-tests were performed to compare mean scores on the continuous AUDIT outcome for the following variables: gender, age group, category of alcohol use, and professional help or medication. At follow-up, the mean for the continuous outcome variable, i.e., perceived success, was compared between hazardous users and other users. Changes in the total AUDIT scores were examined through repeated measures t-tests. Separate analyses were performed for men and women, the different age groups, the various categories of alcohol use at baseline, and the people with or without other professional help or medication.

Next, changes in risk-group-belonging, i.e., changes in category indicated by the AUDIT score between baseline and follow-up, were examined. To see whether the bivariate results, e.g., on gender, were independent of the other covariates, and also to address the issue of discriminating change (i.e., a change in risk-group-belonging) into account, logistic regressions models were used to estimate the odds for the different steps of change in the categories of alcohol use. SAS software was used for all the statistical analyses, including Wald’s chi-square tests for the logistic regression modeling based on Proc Logistic.

Outcome change was based on the possible steps of change in the different categories of alcohol use. No change was coded 0, one step of positive change from a higher category of use to a lower was coded 1, two positive steps 2 (e.g., from dependence to hazardous use), and three steps 3. The corresponding negative steps of change were coded -1, two negative steps -2, and three -3. Thus, the outcome could differentiate between seven different values, ordered from negative to positive.

First, the regressions modeled ORs for the independent variables separately. Second, all variables were entered simultaneously. Lastly, all non-significant variables were excluded from the model with all variables (p < .05), using a backwards stepwise-exclusion procedure.

Chi-square scores supported our proportional odds assumptions, i.e., that the ORs of the independent variables were the same across all the different steps of change.

## Results

Of all the 480 callers to the Help Line during its first calendar year, 47 percent (n = 226) were people seeking support for their own alcohol-related problems (clients). Other callers were relatives of persons with alcohol problems, 31 percent (n = 147). There were also calls on other matters (19%, n = 91), e.g., health care personnel wanting information, prank calls, and wrong numbers.

Table 1 presents the gender and age distributions of all the callers, followed by all the clients. The clients were then separated into those registered as “reactive” and “proactive”. The total number of clients during the study period was 226, of which 147 (65%) were registered for reactive support, and 79 (35%) for proactive support (Table 1). Available mean AUDIT score at baseline, and the number of persons these observations could be based on, are presented in the bottom row of Table 1. The mean AUDIT score at baseline for all available clients was 21.3 points (Table 1), which was similar to the mean scores for reactive clients (21.1 points), proactive clients (21.5 points), and the follow-up sample (20.8 points).

**Table 1:** Callers and clients registered for a reactive or proactive support at the Swedish Alcohol Helpline together with the proactive clients in the 6-months follow-up

	**All callers**	**All clients**	**Reactive clients**	**Proactive clients**	**Follow-up**
**TOTAL**					
Number	479	226	147	79	40
*Men***-%** (n)	40.0 (192)	50.4 (114)	51.7 (76)	48.1 (38)	40.0 (16)
*Women***-%** (n)	60.0 (287)	49.6 (112)	48.3 (71)	51.9 (41)	60.0 (24)
**Mean age**					
years	47.9 (255)	48.6 (168)	47.6 (97)	49.8 (71)	50.2 (39)
SD	16.1	15.2	15.8	14.4	14.0
**AUDIT**					
mean (n)	Nr.	21.3 (120)	21.1 (63)	21.5 (57)	20.8(40)
SD		7.2	7.4	7.0	6.9

Table 2 shows the average AUDIT scores, in total, and by gender, age group, category of alcohol use at baseline, and other professional help or medication. The first column presents mean score for all the 120 clients who responded to the AUDIT questions at baseline. The third column presents mean AUDIT scores for the 40 clients who could be followed up in the pilot study from baseline. This is followed by a fifth column, presenting the AUDIT score for the clients in the pilot study at 6 months follow-up. The final column in Table 2 shows the significance levels for the differences between the two measurement points for the 40 clients comprising the pilot study sample.

**Table 2:** Mean AUDIT scores by subgroup (gender, age, category of alcohol use, and help), for all clients and for the follow-up sample at baseline and follow-up

	**All clients**		**Follow-up clients only**				**Change**^**1**^		
	**Mean baseline AUDIT score (n)**	**SD**	**Mean baseline AUDIT score (n)**	**SD**	**Mean follow-up AUDIT score**	**SD**	**t**	**df**	***p-value***
**Total**	21.3 (120)	7.2	20.8 (40)	6.9	14.3	7.4	5.6	39	.001
**Gender**									
Men	22.5 (57)	7.1	22.3 (16)	7.0	12.6	6.7	4.9	15	.001
Women	20.2 (63)	7.0	19.8 (24)	6.8	15.4	7.8	3.5	23	.002
**Age group**									
15-39	22.5 (36)	5.3	21.6 (9)	5.0	12.0	7.3	5.6	8	.001
40-49	21.0 (24)	7.6	23.3 (10)	8.0	18.3	7.6	1.8	9	.108
50-64	23.8 (33)	7.9	21.4 (13)	7.4	14.7	7.4	2.9	12	.012
65+	16.1 (17)	5.0	14.7 (7)	3.9	9.6	4.2	2.2	6	.068
**Category of alcohol use**									
Hazardous use, 8–15 points^2^	12.6 (26)	2.2	12.5 (11)	2.5	11.6	5.5	0.4	10	.697
Harmful use, 15–19 points	17.6 (32)	1.2	17.9 (9)	1.3	9.8	5.6	4.6	8	.002
Dependence, 20–40 points	26.9 (62)	4.8	26.7 (20)	3.6	17.7	7.6	5.8	19	.001
**Other professional help or medication**									
No	No information		19.3 (15)	5.1	10.5	5.6	4.4	14	.001
Yes	No information		21.7 (25)	7.7	16.5	7.5	3.8	24	.001

^1^Results from the t-tests for repeated measurements in the follow-up sample.

^2^The cut-off was 8 for men and 6 for women.

The proportions of hazardous users, harmful users, and persons dependent on alcohol were similar in the sub-cohort of 40 clients followed up in the pilot study (Sample 2) to those in the baseline assessment, which included all the 120 clients completing the AUDIT (Sample 1); the proportions of hazardous users were 25/120 (20%) and 10/40 (25%), respectively (not shown in the table).

In the follow-up sample, the total AUDIT-score was reduced from 21 to 14 points (see Table 2). The AUDIT scores suggest that men and women did not have significantly different [t(38) = -1.1, p = 0.26] drinking profiles at baseline. There were no significant differences between the age groups, with the exception of the oldest age group, which had a lower mean score than that of the others [15–39 years: t(37) = -0.4, p = 0.69; 40–49 years: t(37) = -1.4, p = 0.18; 50–64 years: t(37) = -0.4, p = 0.68; 65+ years: t(37) = 2.7, p = 0.01].

Both men and women decreased in total AUDIT score significantly from baseline to follow-up (Table 2). Similarly, separate analyses of the age groups indicated that decreases occurred over all ages. The separate analyses of the AUDIT scores for the three different categories at baseline showed that persons with harmful use or dependence decreased their scores significantly, but no significant decrease was detected for hazardous users (Table 2). The analyses also showed that a decrease in AUDIT score among those who received other professional help and those only receiving support from the Helpline were both significant.

Next, we examined changes as transitions between the different categories of alcohol use. At follow-up, 18 (45%) of the 40 clients showed a positive change from a higher category of use to a lower; 18 (45%) showed no change; and 4 (10%) showed negative change from a lower category of use to a higher. Of the 40 clients, 5 (12%) had changed to the non-hazardous group.

Table 3 shows the results of the ordered logistic regressions. In the bivariate analysis, gender, category of use at baseline, and medication were significantly related to change. In Model I, including all the independent variables, i.e., age group, category of use at baseline, medication, and help from friends showed significant relationships. In the final reduced model, Model II, category of use at baseline, medication, and help from friends showed significant relationships. The odds of having made positive changes were significantly higher for persons with a harmful use or dependence at baseline than for people with hazardous use. People who did not take medication (n = 25) to change their alcohol habits had significantly higher odds of having made a positive change than people who did take medication. People who did not receive any help from friends (n = 36) to change their alcohol habits showed significantly higher odds of having made a positive change than people who did receive help from friends.

**Table 3:** Odds Ratios (ORs) for positive change in ordered logistic regression models for the different variables (n = 40)

		**Bivariate**		**Model I**		**Model II**_**reduced**_	
**Independent variables**		**OR**	**95% CI**	**OR**	**95% CI**	**OR**	**95% CI**
**Gender**							
	Women	ref.		ref.		-	
	Men	5.69^**^	1.59-20.33	2.63	0.54-12.84	-	
**Age group**	15-39						
	40-49	ref.		ref.		-	
	50-64	.35	0.07-1.87	.10^*^	0.01-0.86	-	
	65+	.23	0.05-1.16	.28	0.04-2.22	-	
		.57	0.10-3.44	1.98	0.16-23.94	-	
**Category of alcohol use at baseline**							
	Hazardous use^1^	ref.		ref.		ref.	
	Harmful use	18.3^**^	2.41-138.49	21.6^*^	1.69-276.06	19.1^*^	2.01-181.37
	Dependence	11.4^**^	1.91-68.63	128^***^	8.21-999.99	32.0^**^	3.93-259.77
**Other professional help**							
	No	ref.		ref.		-	
	Yes	.56	0.18-1.77	.80	0.15-4.25	-	
**Medication**							
	No	ref.		ref.		ref.	
	Yes	.19^*^	0.05-0.71	.08^**^	0.01-.50	.12^**^	0.03-0.54
**Help** from friends							
	No	ref.		ref.		ref.	
	Yes	.40	0.06-2.87	.12	0.01-1.98	.09^*^	0.01-0.96
from family							
	No	ref.		ref.		ref.	
	Yes	4.29^*^	1.21-15.16	2.35	0.48-11.47	3.85^2^	0.93-15.85
**Treatment**							
	Reduce the no. of times	ref.		ref.		-	
**Goals**							
	Reduce the amount	.11	0.01-1.06	.14	0.01-3.36	-	
	To be able to control	.60	0.14-2.55	.72	0.12-4.21	-	
	Complete or period stop	.71	0.15-3.30	1.00	0.16-6.07	-	
	Else	.20	0.01-10.55	.01	0 .00-1.71	-	

^*^p < .05 ^**^p < .01 ^***^p < .001

^1^The cut-off was 8 for men, but 6 for women. The only person with non-hazardous alcohol use at baseline was included in the reference group.

^2^Help from the family (n = 14) was left in the model as a control, as it was confounded with help from friends.

Not shown are the estimated intercepts (in ORs) for the change model. In the last model the odds for all steps of change differed significantly from each other. In the model without independent variables, all steps except the step from negative change to no change were significant (p < 0.05).

Assessment of the primary personal goals revealed that 72.5% (29/40) of the follow-up clients had primarily wanted to “gain control over their drinking”, and approximately 22.5% (9/40) stated that they wanted to “quit drinking completely” as a primary goal. Two clients (5%) were not able or willing to formulate primary goals for contacting the Helpline (not shown in the tables).

Perceived success at follow-up of achieving initial goals indicated partial success. The average score was 6.2 (SD = 2.8), and there was no significant difference (t(39) = 0.8, p = 0.45) between the hazardous users (M = 5.6, SD = 3.1) and the others (M = 6.4, SD = 2.6) in this respect (not shown in the tables).

## Discussion

Although not conclusive, the results from the present study represent a first step in describing professionally run telephone helplines as a primary care option for people with alcohol-related problems. Of the 480 callers to the Alcohol Helpline during its first year, about half were clients, whereas one-third wanted to discuss the alcohol behaviors of relatives. As a consequence, as time has passed, the explicit aim of the Helpline has been extended to include other help, e.g., help to relatives and other people close to persons with alcohol-behavior issues. In comparison with other treatment options in Sweden, the telephone line reaches a high proportion of women [52]. Of the 120 clients with baseline AUDIT assessment, a substantial proportion (n = 25, 20%) were categorized as hazardous alcohol users on the AUDIT scale, indicating that the service may have the potential to reach people at early stages of alcohol abuse.

In the general Swedish population, 14 percent of men and 13 percent of women have been estimated to have hazardous alcohol use, while four percent of the men and one percent of the women may be classified as alcohol dependent, i.e., an AUDIT score of 15+ for men, and 13+ for women [33]. The Helpline’s clients have an average AUDIT score of 21 points, which equals the score of patients at a psychiatric emergency care unit [48]. In a US sample of alcohol-dependent individuals entering outpatient care, the average AUDIT score was 26 points, and the distribution was 4, 11 and 85 percent for hazardous use, harmful use, and dependence, respectively [51]. The corresponding distribution in the present study was 21, 27, and 52 percent, respectively. In comparison with web-based support [53,54], or screening at primary care receptions [55], a clientele with more severe alcohol use seems to be reached by the Helpline.

The decrease in AUDIT score over time noted in the high-consumer group fits the idea that “treatment effects” are usually stronger than “prevention effects”. Thus, those with the initially highest problems have the highest scope for improvement, whereas those with the lowest problems have the lowest scope for change. It also fits a regression-to-the-mean tendency, meaning here that heavy consumers are more likely to decrease their drinking than average consumers [56]. The design of the current study does not enable us properly to evaluate these different explanations of change in AUDIT score.

The population of callers may also have changed over time, since information about the Helpline has become more widespread. Such changes in the study population limit the generalizability of our results. Our findings indicate that people with hazardous alcohol use are a difficult target group to reach. People with less severe drinking problems seem to be less likely to seek treatment or support.

A majority of the clients chose reactive services. Consequently, the pilot-study sample (Sample 2) comprises a select group of clients who accepted being registered as proactive. Although there were no differences in AUDIT scores between the proactive and reactive clients, or between the whole sample and the follow-up at baseline, it remains unclear how selection might have biased our results. The clients choosing proactive support may, for example, have been highly motivated people who were about to change anyway, i.e., that change would come with non-regarding treatment Also, the observed change in the follow-up group was not unusual size [30]. Studies of brief interventions in primary care of people with high alcohol use have consistently reported decreases in both treated and untreated (control) individuals [57]. Further, studies also suggest an effect of the screening procedure [58].

The counseling protocol in the present study appears to have been helpful and, although the present pilot study is not conclusive, the potential and novelty of the approach deserve further investigation. One encouraging finding from the pilot study is that many clients experience that they have achieved their personal goals for contacting the Helpline. Nevertheless, it remains unclear whether the Helpline’s treatment is effective.

Apparently, the counseling offered by the Alcohol Helpline is often conceived as a supplementary support when people are trying to change their alcohol habits. According to the follow-up, more than half of the clients had received additional professional help to modify their alcohol habits during the follow-up period. This may partly be due to the helpline support protocol which encourages severe alcohol users also to seek other forms of help. Unfortunately, we can only speculate on this issue, since it was not systematically assessed in the follow-up interview.

In an intervention concerning university students, the AUDIT score in the high-risk group was reduced from 11.5 to 7.5 after 6 months [49]. Our follow-up indicates a general change of about the same relative size, but from higher initial AUDIT scores.

In the present pilot study, no significant behavior change was noted for hazardous users during the study period. A longer follow-up period may be needed to capture change in this group of hazardous alcohol users. Also, it is possible that the helpline clients with hazardous use may differ from the corresponding target group in the general population. The AUDIT instrument discriminates alcohol-related behavior and consequences only during the previous year. It would have been desirable also to measure alcohol problems earlier in life, since clients of this kind may have been utilizing the service as an aid to preventing relapse into riskier alcohol habits. Thus, whether the Helpline prevents hazardous drinkers from progressing into more severe drinking is unclear.

Using medication was associated with lower odds for making positive change. Possibly, the use of medicines can be seen as a marker of more serious or long-lasting alcohol problems. On the other hand, such an interpretation goes against other results, namely that positive change is primarily found among persons with harmful use or dependence. The small sample size and a lack of randomization inhibit the interpretation of our results.

Most randomized control studies of telephone counseling have indicated positive treatment results in health care circumstances [10-16,20,21,23,24], and also elsewise [17-19]. In one study, primary care patients received up to six episodes of telephone counseling after systematic screening [13]. The results showed that the intervention group exhibited greater decline in risky drinking days compared with the controls who simply received an information pamphlet. In another study, all patients in 81 German general practices were screened for alcohol use disorder [14]. Eligible participants received computerized interventions plus a maximum of four brief telephone counseling sessions. A small to a medium effect size of the treatment was found for the intervention group compared with controls. But no difference was found between a group of patients who received stepped care, i.e., computerized intervention plus up to three 40-min telephone based counseling episodes (depending on the success of the previous intervention),and a group who received care equaling the maximum amount of stepped care.

Although non-randomized descriptive studies, such as the present one, are useful when priorities for further investigations are identified or when randomized trials are unethical [59-63], a future study of treatment effects in a telephone helpline setting needs to include a randomized control group. A control group would capture the naturally occurring change in this particular self-selected group of people.

### Implications for practice

To our knowledge, earlier studies have not investigated telephone helplines as a primary care option for alcohol users. The helpline is to a large extent reliant on people’s own identification of their health problems, and focuses support primarily on the clients’ own personal goals, which is in accordance with the essence of motivational interviewing.

Since alcohol problems often remain hidden from health care [64], a helpline where you can remain anonymous, may provide a feasible alternative and/or function as a stepping stone towards a more clinic-based support.

## Competing interests

There are no direct competing financial interests between the authors and the Swedish Alcohol Helpline. However, during the time of the data collection the authors KA and LN salaries were partly financed by the same unit at Stockholm County Council Health Services (SCCHS) responsible for the development of the Swedish Alcohol Helpline, while author ARH was founded by another unit at SCCHS during the same period.

## Authors’ contributions

Author KA wrote the draft and did the statistical analysis. The authors LN and ARH were responsible for the data collection and the design behind the pilot study. The final draft has been overseen and contributed to by all authors. All authors read and approved the final manuscript.
